# Novel frameshift variants expand the map of the genetic defects in IRF2BP2

**DOI:** 10.3389/fimmu.2023.1279171

**Published:** 2023-10-09

**Authors:** José María García-Aznar, Emilia Maneiro Pampín, Maite García Ramos, María José Acuña Pérez, Nerea Paz Gandiaga, Laura Minguell Domingo, Olga Calavia, Pere Soler-Palacin, Roger Colobran, Erika M. Novoa Bolívar, Javier Gonzalo Ocejo Vinyals

**Affiliations:** ^1^ Department of Immunology, Health in Code, A Coruña, Galicia, Spain; ^2^ Genetics Division, Universitary Hospital Marqués de Valdecilla, Santander, Canatabria, Spain; ^3^ Pediatrics Division, Universitary Hospital Arnau de Vilanova, Lleida, Catalonia, Spain; ^4^ Pediatrics Division, Hospital Joan XXIII, Tarragona, Catalonia, Spain; ^5^ Pediatric Infectious Diseases and Immunodeficiencies Unit, Children’s Hospital, Barcelona, Catalonia, Spain; ^6^ Infection and Immunity in Pediatric Patients Research Group, Vall d’Hebron Research Institute (VHIR), Barcelona, Catalonia, Spain; ^7^ Immunology Division, Vall d’Hebron University Hospital (HUVH), Barcelona, Catalonia, Spain; ^8^ Translational Immunology Research Group, Vall d’Hebron Research Institute (VHIR), Barcelona, Catalonia, Spain; ^9^ Department of Clinical and Molecular Genetics, Vall d’Hebron University Hospital (HUVH), Barcelona, Catalonia, Spain; ^10^ Department of Cell Biology, Physiology and Immunology, Autonomous University of Barcelona (UAB), Barcelona, Catalonia, Spain; ^11^ Immunology Division, Universitary Hospital Virgen de la Arrixaca, Murcia, Spain; ^12^ Immunology Division, Universitary Hospital Marqués de Valdecilla, IDIVAL, Santander, Cantabria, Spain

**Keywords:** IRF2BP2, CVID, colitis, primary immunodeficiency, loss-of-function mutations

## Abstract

**Background:**

At present, the knowledge about disease-causing mutations in IRF2BP2 is very limited because only a few patients affected by this condition have been reported. As previous studies have described, the haploinsufficiency of this interferon transcriptional corepressors leads to the development of CVID. Very recently, a more accurate phenotype produced by truncating variants in this gene has been defined, manifesting CVID with gastrointestinal inflammatory symptoms and autoimmune manifestations.

**Methods:**

We analyzed 5 index cases with suspected primary immunodeficiency by high throughput sequencing. They were submitted for a genetic test with a panel of genes associated with immune system diseases, including IRF2BP2. The screening of SNVs, indels and CNVs fulfilling the criteria with very low allelic frequency and high protein impact, revealed five novel variants in IRF2BP2. In addition, we isolated both wild-type and mutated allele of the cDNA from one of the families.

**Results:**

In this study, we report five novel loss-of-function (LoF) mutations in IRF2BP2 that likely cause primary immunodeficiency, with CVID as more frequent phenotype, variable expression of inflammatory gastrointestinal features, and one patient with predisposition of viral infection. All identified variants were frameshift changes, and one of them was a large deletion located on chromosome 1q42, which includes the whole sequence of IRF2BP2, among other genes. Both *de novo* and dominant modes of inheritance were observed in the families here presented, as well as incomplete penetrance.

**Conclusions:**

We describe novel variants in a delimited low-complex region, which may be considered a hotspot in IRF2BP2. Moreover, this is the first time that a large CNV in IRF2BP2 has been reported to cause CVID. The distinct mechanisms than LoF in IRF2BP2 could cause different phenotype compared with the mainly described. Further investigations are necessary to comprehend the regulatory mechanisms of IRF2BP2, which could be under variable expression of the disease.

## 1 Introduction

The IRF2BP (interferon regulatory factor-2 binding protein) family is composed of two members, IRF2BP1 and IRF2BP2, characterized by a C-terminal repression domain. These proteins have been reported to act as nuclear transcriptional corepressors that are activated during IRF2 binding, which induces a conformational change in the DNA binding domain and the C-terminal region of IRF2 (1). This gene is a co-repressor of IRF2, decreasing JAK-STAT5 signaling induced by IL-2 in naïve CD4+ T cells and, regulating the CD25 and CD69 markers and other factors upon the stimulation of IL2 (2). Two different isoforms have been established: the long isoform, isoform A (NM_182972.3; NP_892017.2), which spans 587 amino acids, and isoform B (NM_001077397.1; NP_001070865.1), of 571 amino acids. It is organized into two exons which contains the coding sequence for one zinc finger RING-type domain located in the C-terminal region and a nuclear localization signal, to name a few relevant motifs of the protein. Recently, Pastor et al. (2021) described a third isoform, isoform C, with a non-defined CDS of 163 amino acids, whose function is still unknown (3).

One of the main targets of its negative regulator function is the NFAT gene, a nuclear protein that regulates cell cycle progression and cell differentiation (4). However, IRF2BP2 also modulates other pathways that do not depend on IRF2. Besides its repressor activity, IRF2BP2 also stimulates the expression of some genes related to proliferation, migration, and inflammatory processes, such as the VEGFA or JAK–STAT signaling pathways (5, 6). Also, its expression has been proposed to participate in lipid metabolism with an important role in inflammation in atherosclerosis (7). This protein has also been found to be involved in non-immunological processes. For this instance, IRF2BP2 overexpression suppresses osteoclast differentiation and enhances osteoblast differentiation, thus regulating bone homeostasis (8).

During this genomic era, common variable immunodeficiency (CVID) is considered a heterogeneous group of conditions spanning penetrant monogenic forms and also oligogenic and polygenic causes. Almost 80 genes associated with PID have been related in some way with manifestations of clinical diagnosis of CVID, showing the numerous pathways involved in these disorders (9). According to the European Society for Immunodeficiencies (ESID), the clinical criteria to design CVID as clinical diagnosis must include (i) at least one of following: increased susceptibility to infection, autoimmune manifestations, granulomatous disease, unexplained polyclonal lymphoproliferation or affected family member with antibody deficiency; (ii) marked decrease of IgG and marked decrease of IgA with or without low IgM levels; and (iii) at least one of the following: poor antibody response to vaccines or low switched memory B cells. In addition, these patients must not have secondary causes of hypogammaglobulinemia, evidence of profound T-cell deficiency and the diagnosis should be established after fourth year of life (10).

Genetic defects with loss-of-function in IRF2BP2 have been candidates to be associated with autosomal dominant CVID during many years. A few sporadic families have been reported in the scientific literature, suggesting that disease-causing mutations in this gene are very rare. The first disease-causing mutation in the IRF2BP2 gene associated with CVID was the missense variant p.Ser551Asn, described in 2016 (11). Very recently, Palmroth et al. (6) defined a more accurate phenotype with inflammatory manifestations associated with loss of function of IRF2BP2 resulting from the presence of a truncating mutation. To date, three truncating variants have been reported to be associated with this phenotype: one deletion producing a frameshift mutation, p.Ala209Glnfs*31 (6); one insertion, p.Gln536delins* (12); and one nonsense mutation, p.Gln540*, located in the C-terminal region of IRF2BP2 (13).

In this article, we describe four new variants in IRF2BP2 predicted to cause LoF and one large deletion encompassing the whole IRF2BP2 gene from a Spanish cohort.

## 2 Materials and methods

### 2.1 High throughput sequencing

Five index cases were analyzed using a High-Throughput Sequencing (HTS) protocol. DNA from the blood samples of the probands was extracted and purified on QIAsymphony SP (Qiagen). Library preparation for sequencing was carried out using the Agilent SureSelect Library Preparation Kit for Illumina paired-end multiplexed sequencing according to the manufacturer’s instructions. We enriched the regions of interest using a SureSelect Probe Kit (Agilent) that selectively captures the coding regions and adjacent intronic areas +/- 50 base pairs of the exons of 549 genes selected for immunological disease (see **Supplementary Material**). Cluster preparation was performed on the cBot (Illumina, San Diego, CA, USA) device. Bioinformatics analysis was done with an end-to-end in-house pipeline developed by Health in Code (A Coruña, Spain).

### 2.2 Sanger sequencing DNA

After the identification of the candidate variants in the probands, we confirmed the finding by Sanger sequencing in the three patients and their family members, using a specific design to screen for changes in exon 1 of *IRF2BP2*. Original primers for PCR amplification of the target region in exon 1 were applied in order to avoid a repeat masker sequence using the forward primer 3’GGACTTCACCGAACCCGTCT 5’ and the reverse primer 5’GGCTGCTGCCGAAGTCAGAG 3’. The primers for Sanger sequencing used to read the sequence of the variant detected by NGS were the forward primer 3’AACGGCTTCTCCAAGCTAGA 5’ and the reverse primer 5’GAATGTGCTGGGAAAGGAAA 3’.

### 2.3 CGX-HD array

CGX-HD array technology with an average resolution of the genome of about 40 Kb was used to confirm the large CNV affecting the chromosome region 1q42.2 in family 4. This approach examined 980 genes associated with genetic diseases. The data were interpreted with Genoglyphix software.

### 2.4 RNA isolation, RT-PCR and cDNA synthesis

The extraction of total RNA was performed from 2.5 mL of whole blood collected into PAXgene RNA Tubes using the Maxwell 16 LEV simplyRNA Tissue Kit (Promega) on the Maxwell 16 Instrument (Promega), following the manufacturer’s protocols. The purified total RNA samples were quantified using a NanoDrop Spectrophotometer (Thermo Fisher Scientific, UK), with average yields ranging between 57 and 158 ng/μL. For reverse transcription, 600 ng of the purified total RNA was utilized, and the SuperScript™ VILO™ cDNA Synthesis Kit (Invitrogen) was employed following the manufacturer’s protocols.

To verify the cDNA synthesis, the ribosomal protein L19 (RPL19) transcript (NM_000981.4) was employed as an endogenous internal control. The RPL19 transcript was amplified using the GC-RICH PCR System dNTPack (Roche) following the manufacturer’s protocols, and the forward primer 5’-CGGCCGCAGCCATGAGTAT-3’ (located at the exon 1-exon 2 junction) and the reverse primer 5’-GGCTGTGATACATGTGGCGA-3’ (located at the exon 4-exon 5 junction) were used to amplify an expected product of 378 bp. A standard thermocycler program with 30 cycles was used (95°C-3 min, 95°C-30 s, 60°C-30 s, 72°C-30 s and 72°C 1 min).

### 2.5 RT-PCR assay of IRF2BP2 endogenous expression

The PCR amplification of IRF2BP2 transcript 2 (NM_001077397.1) was carried out using the GC-RICH PCR System dNTPack (Roche) following the manufacturer’s protocol. The amplification was performed with the forward primer F1 (5’-TCGAGTTCGTCATCGAGACGGC-3’) and the reverse primer 1 (5’-TGCTGCCGAAGTCAGAGCCGAGG-3’), both positioned at exon 1 flanking the c.217_244del variant (263bp). A standard thermocycler program with 30 cycles was used (95°C-3 min, 95°C-30 s, 60°C-30 s, 72°C-30 s and 72°C 1 min). Additionally, an allele-specific PCR assay targeting the c.991C>T polymorphic site, located 10 nucleotides upstream of the exon1-exon2 junction, was designed. The sequence of the reverse primers was 5’-TCTTGCAACTGCAGTCA[G/A]GGCCGG-3’ (RWW and RWT) and the forward primer F1 (5’-TCGAGTTCGTCATCGAGACGGC-3’) was used for this assay for an expected amplification product of 863 bp. A standard thermocycler program with 35 cycles was used (95°C-3 min, 95°C-20 s, 66°C-20 s, 72°C-45 s and 72°C 7 min).

Quantification and sizing of RT-PCR products were performed using the Agilent 4200 TapeStation system and D1000 ScreenTape according to the manufacturer’s protocol.

### 2.6 Sanger sequencing of RT-PCR products

The RT-PCR products underwent an enzymatic clean-up process prior to sequencing. This clean-up involved the use of exonuclease I and thermosensitive alkaline phosphatase (Thermo Fisher Scientific), following the manufacturer’s protocols. For sequencing, both forward and reverse reactions were carried out using the Big Dye Terminator v3.1 Cycle Sequencing kit (Applied Biosystems) and the same primers employed in the PCR, with the exception of the 5’-AGGGTCGCTCCCCAAGCCGCC-3’ (FΔdel28), primer, which was specifically designed to overlap the 28-bp deletion, and the primer 5’-AACGGCTTCTCCAAGCTAGA-3’ (F2), which was designed to align in the middle of exon 1. Following cycle sequencing, the resulting products were purified using the BigDye XTerminator Purification kit (Applied Biosystems) and subjected to capillary electrophoresis using the ABI3730 DNA Analyzer (Applied Biosystems). The Sanger chromatograms were visualized and analyzed using Chromas software, and the ABI files were then converted to PDF files.

### 2.7 Patient consent for publication

Written informed consent was obtained from all patients for the genetic analyses and anonymization.

## 3 Results

### 3.1 Patient description

The first case (1.1), from family 1, is a 45-year-old woman diagnosed with CVID 23 years prior. Her immunophenotype revealed a decreased and defective isotype switching of B-cells together with IgG, IgA, and IgM hypogammaglobulinemia. At the age of 16, she had been diagnosed with insulin-dependent diabetes mellitus together with hidradenitis suppurativa. Recurrent respiratory infections with lingula and left lower lobe bronchiectasis and gastrointestinal infections are present since childhood, and in addition, she developed severe colitis. She was receiving immunoglobulin replacement therapy. The patient’s father (1.2) had a history of recurrent pneumonia and hypogammaglobulinemia. The patient’s mother died due to cardiovascular problems. Throughout her life, she did not suffer from notable infectious agents.

The second case (2.1), from family 2, is a 31-year-old male with severe hypogammaglobulinemia and Crohn’s like enteropathy. This ileal Crohn disease was unresponsive to standard therapies, requiring ustekinumab and ileal resection at the age of 30. His parents were healthy and without remarkable clinical history.

The third case (3.1), from family 3, is a 26-year-old male with CVID diagnosed 12 years prior due to recurrent otitis media and pneumonia. He was of Romani ethnicity, and his parents were consanguineous (first cousins). He presented with severe bronchiectasis (upper right lobe, medium lobe, lingula lobe and pulmonary bases). In addition, no other clinical symptoms were present. His father (3.2) was asymptomatic with mildly low switched memory B-cells.

The fourth case (4.1), from family 4, is a 15-year-old male with a history of recurrent bronchiectasis and persistent hypogammaglobulinemia. Moreover, the patient showed features of hypotonia, hyperlaxity and pectus carinatum, which were suspected to be consistent with Ehlers Danlos syndrome, which was not confirmed according to the negative genetic test.

The fifth case (5.1), from family 5, is a 13-year-old male born in a non-consanguineous family after *in vitro* fertilization using a donor egg. In early childhood, he presented with recurrent laryngitis, bronchitis and one bronchopneumonia at age10 months. At the age of 3 years, after the first dose of the varicella vaccine, he suffered a side effect with an episode of right hemiplegia with headache lasting 15 minutes (with no facial paralysis or gaze deviation) that he was completely recovered. At the age of 7, he developed a mild chickenpox and ten days later he had an atypical pneumonia. Finally at 13-year-old, he was admitted to the hospital due to a chickenpox meningitis with a few vesicular skin lesions. Diagnosis was made by PCR in cerebrospinal fluid. MRI was normal and he recovered after 14 days of acyclovir treatment without any side effects. Basic immunological study was normal, including immunoglobulins in the normal range.

The summary of clinical information of affected patients is shown in **Table 1**.

**Table 1:** Clinical and immunological features of affected patients reported in this study.

Patients	IgG (mg/dL)	IgA (mg/dL)	IgM (mg/dL)	B-cell immunephenotype (CD19+IgD-CD27+ switched memory)	T-cell immunephenotype	Response to vaccines	Clinical manifestations	Treatment
1.1	IgG=477	0,63	43,7	0,71 (9.2 - 18.9)	normal	Poor	Respiratory infections, Bronchiectasis, Gastrointestinal infections, Severe colitis, DM, hidradenitis suppurativa	Ig replacement therapy
1.2	IgG1 = 309 IgG2 = 3,8 IgG3 = 8,9 IgG4<0,4	<2,08	92,11	1,47 (9.2 - 18.9)	normal	N/A	Recurrent pneumonia	Ig replacement therapy
2.1	low*	low*	low*	N/A	N/A	Poor	Croh’s disease, Secondary spondylarthritis	Ig replacement therapy; immunosupression; Ileal resection
3.1	IgG1 = 96,3 IgG2 = 10,9 IgG3 = 0,68 IgG4 = 0,32	<6,2	<6,4	0,88 (7.2 - 12.7)	normal	N/A	Recurrent pneumonia, Bronchiectasis, Otitis media infections	Ig replacement therapy
3.2	IgG1 = 359 IgG2 = 293 IgG3 = 39,4 IgG4 = 0,7	197,44	46,48	7.18 (9.2 - 18.9)	N/A	N/A	Asymptomatic	no
4.1	IgG1 = 163 IgG2 = 2 IgG3 = 34 IgG4 = 6	<0.7	10	N/A	N/A	Poor	Bronchiectasis,hypotonia, hyperlaxity, pectus carinatum	Ig replacement therapy
5.1	IgG1 = 584 IgG2 = 245,7 IgG3 = 86 IgG4 = 33,6	N/A	N/A	12,61 (6-16)	Increased CD8+ CD45RA+ CCR7-	Poor	Recurrent laryngitis, bronchitis and one bronchopneumonia, chickenpox meningitis; increase pDC	

Clinical and immunological data from affected patients with novel mutations in IRF2BP2. The level of Immunooglobulins and B-cell/T-cell subpopulations were indicated at the moment of diagnosis. N/A, non-available information. All patients with the exception of 3.2 and 5.1 received Ig replacement therapy. * The immunological data from patient 2 were obtained from old clinical history. The current values were disturbed due to immunosuppressed treatment since adolescence.

### 3.2 Loss-of-function variants in IRF2BP2

In the five reported patients of this study, a truncating germline variant in the *IRF2BP2* gene was detected, all of them absent in the control population of the gnomAD database. The patient 4.1 carried a large deletion involving the whole *IRF2BP2* gene. Molecular details were shown in **Table 2**. Special attention deserves the fifth patient, which harbored a frameshift variant likely causing the extension of the protein sequence, thus its behavior seems to be different than the other frameshift variants which caused a premature stop codon of the translation. Each one of five-novel frameshift variants in IRF2BP2 were detected by fulfilling the criteria of low allelic frequency (<0,01% in gnomAD), high protein impact (non-synonymous, nonsense, frameshift, indels and synonymous or intronic variants with potentially effect in the splicing process) and reliable quality parameters (according to the score of the genotypers used). By using these criteria each truncating variant was the first time observed from a cohort of 2111 patients studied by the same methodology.

**Table 2:** Molecular features of patients reported in this study.

Family	Patient	Variant position	Zygosity	Allele frequency in gnomAD	Pathogenicity	ACMG criteria
1	1.1	c.217_244del; p.Pro73Serfs*72	Het	0%	Pathogenic	PVS1, PM2, PP4
1	1.2	c.217_244del; p.Pro73Serfs*72	Het	0%	Pathogenic	PVS1, PM2, PP4
2	2.1	c.324_325insAGGCGGCCCCG; p.Ala110Argfs*48	Het	0%	Pathogenic	PVS1, PS2 PM2, PP4
3	3.1	c.314_324del; p.Glu105Alafs*24	Het	0%	Likely pathogenic	PVS1, PM2, BS4
4	4.1	Del 1q42.2-42.3	Het	0%	Pathogenic	PVS1, PS2 PM2, PP4
5	5.1	c.1754_1757del; p.Arg585Thrfs*12	Het	0%	VUS	PM2, BS4

PVS1: Null variant in a gene where LoF is a known mechanism of disease; PS2: De novo in a patient with the disease and no family history.; PM2: Absent from controls; PP4: Patient’s phenotype or family history is highly specific for a disease with a single genetic etiology; BS4: Lack of segregation in affected members of a family.

**Figure 1:**
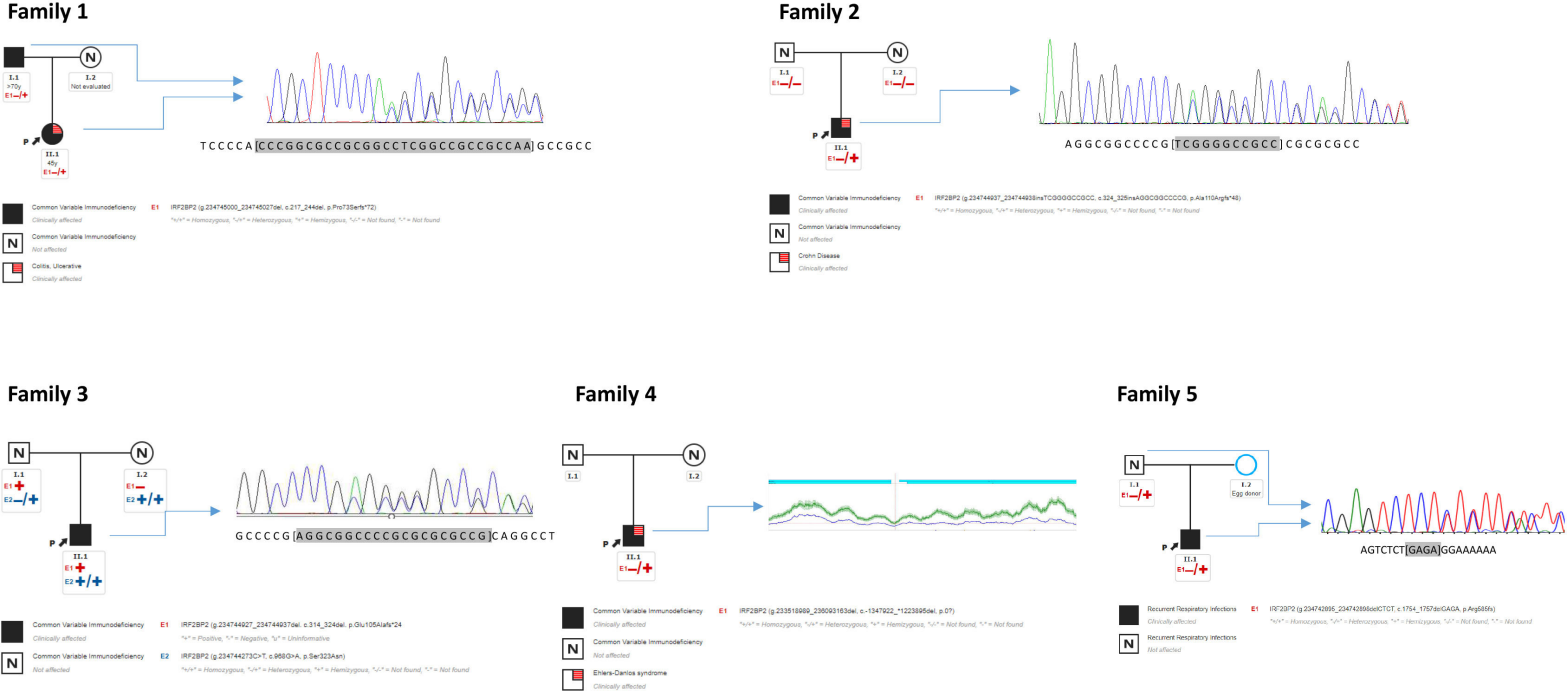
Pedigree of families 1, 2, 3, 4, 5. Proband from family 1 (1.1 “II.1”) harbors one heterozygous deletion in *IRF2BP2* inherited from an affected father (1.2 “I.2”). Proband from family 2 (2.1 “II.1”) harbors one *de novo* insertion absent in his parents. Proband from family 3 (3.1 “II.1”) harbors one heterozygous deletion inherited from an asymptomatic father. Proband from family 4 (4.1 “II.1”) harbors one heterozygous large deletion. Proband from family 5 (5.1 “II.1”) harbors one heterozygous deletion inherited from an asymptomatic father. Sanger sequencing of DNA are shown beside pedigree. CNV from coverage analysis belonging NGS sequencing from family 4 is shown beside its pedigree. CNV was confirmed by array CGX-HD, see **Figure 2**. *“I” = first generation; “II” = second generation*.

The patient from family 1 harbored a candidate heterozygous variant in the *IRF2BP2* gene that was thought to be the cause for the disorder (c.217_244del; p.Pro73Serfs*72). This change is a deletion of 28 nucleotides with GC content of 92.6%, affecting the first 73 amino acids of the protein. In silico prediction prognoses the introduction of a premature stop codon of the translation in the residue 145, which would lead to the expression of an aberrant protein that would lack 75% of the original protein sequence, including part of exon 1 and the whole exon 2. HTS alignment analysis showed a bad quality of the studied region; therefore, we proceeded to employ Sanger sequencing to test for this mutation, which was confirmed. The father of the index case was also affected by a similar condition, carrying the same variant and thereby confirming the autosomal dominant inheritance of the variant (**Figure 1**).

The same procedure was used to evaluate the patient from family 2, detecting in this case an heterozygous insertion of 11 nucleotides with high GC content in the *IRF2BP2* gene (c.324_325insAGGCGGCCCCG; p.Ala110Argfs*48). Similarly, to the previous variant, the in silico prediction prognoses the introduction of a premature stop codon of the translation in the residue 168, which would lead to the expression of an aberrant protein that would lack 71% of the original protein sequence. The HTS results for this variant also showed low-quality parameters. This sample was also submitted for Sanger sequencing, which confirmed the presence of the variant. The healthy parents of this case were negative for this mutation, revealing that this was a *de novo* event in the proband (**Figure 1**).

The proband from family 3 harbored a heterozygous deletion of 11 nucleotides in the *IRF2BP2* gene located in the same region as the variant in the above case (c.314_324del; p.Glu105Alafs*24). This variant was inherited from an apparently asymptomatic father (**Figure 1**). For this instance, the in silico prediction prognoses the introduction of a premature stop codon of the translation in the residue 129, which would lead to the expression of an aberrant protein that would lack 78% of the original protein sequence. Besides, this patient carried a rare homozygous variant in *IRF2BP2* (c.968G>A; p.Ser323Asn), also located in exon 1. The parents were found to be homozygous and heterozygous for the variant, respectively, as well as an asymptomatic sister was also heterozygous. This variant was present in 32 heterozygous individuals from the gnomAD database, with bioinformatics predictors of protein damage showing contradictory results and indicating both tolerated and deleterious effects. According to the ACMG criteria, this missense variant is predicted to be benign.

**Figure 2:**
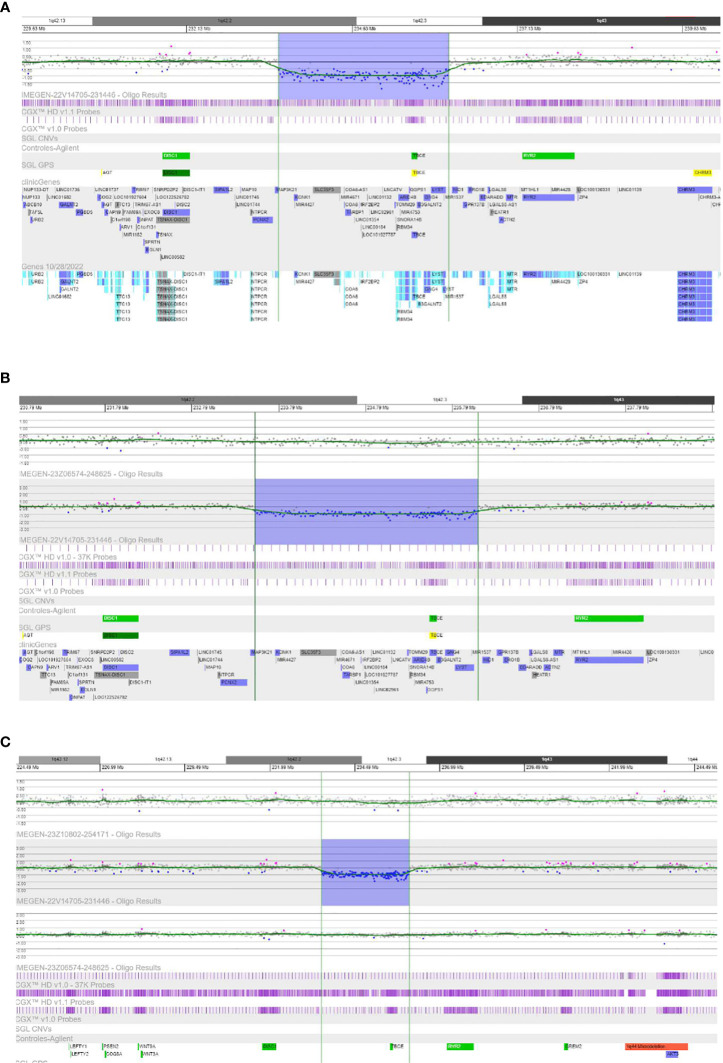
Results of CGX-HD Array (4*180K). **(A)** The probes located in the region 42.2-42.3 from chromosome 1q showed reduced signal by about 50% compared with controls, consistent with a heterozygous deletion of this fragment in the index case (patient 4.1). **(B)** The probes located in the region 42.2-42.3 from chromosome 1q did not show significant alteration of the signal, consistent with the absence of the deletion in the mother of patient 4.1. **(C)** The probes located in the region 42.2-42.3 from chromosome 1q did not show significant alteration of the signal, consistent with the absence of the deletion in the father of patient 4.1.

The proband from family 4 harbored a large deletion encompassing several genes located on chromosome 1q, band 42.2. The following genes were affected by the heterozygous deletion: *ARID4B, B3GALNT2, COA6, GGPS1, GNG4, IRF2BP2, KCNK1, LINC00184, LINC01132, LINC01354, LOC101927765, LOC101927787, LOC101927851, LYST, MAP3K21, MIR4427, MIR4671, MIR4753, RBM34, SLC35F3, SNORA14B, TARBP1, TBCE*, and *TOMM20.* Only *IRF2BP2* has been associated with an autosomal dominant genetic disorder that would be consistent with heterozygous loss of the fragment. This deletion was identified by coverage CNV analysis of data from high throughput sequencing, and was later confirmed by the array (**Figure 2**). The parents were screened for this CNV, with absence of the deletion; therefore, it was concluded to be a *de novo* event in the proband.

The patient from family 5 harbored a heterozygous deletion of four nucleotides in the *IRF2BP2* gene located at the end of exon 2 (c.1754_1757del; p.Arg585Thrfs*12). In fact, this variant is very close to the end of the protein, affecting residue 585 out of 587, causing a frameshift, and adding an additional stretch of amino acids beyond the original STOP codon. This makes the mutant protein longer than the *wild type* (595 vs 587 amino acids respectively). This variant was not found to be reported in the gnomAD database. The variant was confirmed by Sanger sequencing and family screening showed that the *IRF2BP2* variant was inherited from the patient’s apparently asymptomatic father.

In view of the results, observing several large indels in the exon 1 and due to the complexity in the analysis of the region, we explore the content of CGs and complexity by using the BLAST tool (Basic Local Alignment Search Tool) from NCBI and an internal GC content calculator. We identified a low complexity region encompassing c.218_458 and a high GC content (>80%) from c.163_544. Therefore, we consider a low-complex with high GC content region in the exon 1 of IRF2B2 located in c.163_834 (p.54_278).

### 3.3 RT-PCR analysis of endogenous IRFBP2 expression

We conducted an RT-PCR analysis to assess the expression of endogenous RNA-IRFBP2 in the blood samples in Family 1 (proband’s father and index case). Since the variant is located in exon 1, in the vicinity of the translation initiation codon, we initially designed target-specific primers positioned upstream of the predicted premature termination codons (PTCs) to verify the detection of transcripts carrying the c.217_244del variant.

**Figure 3:**
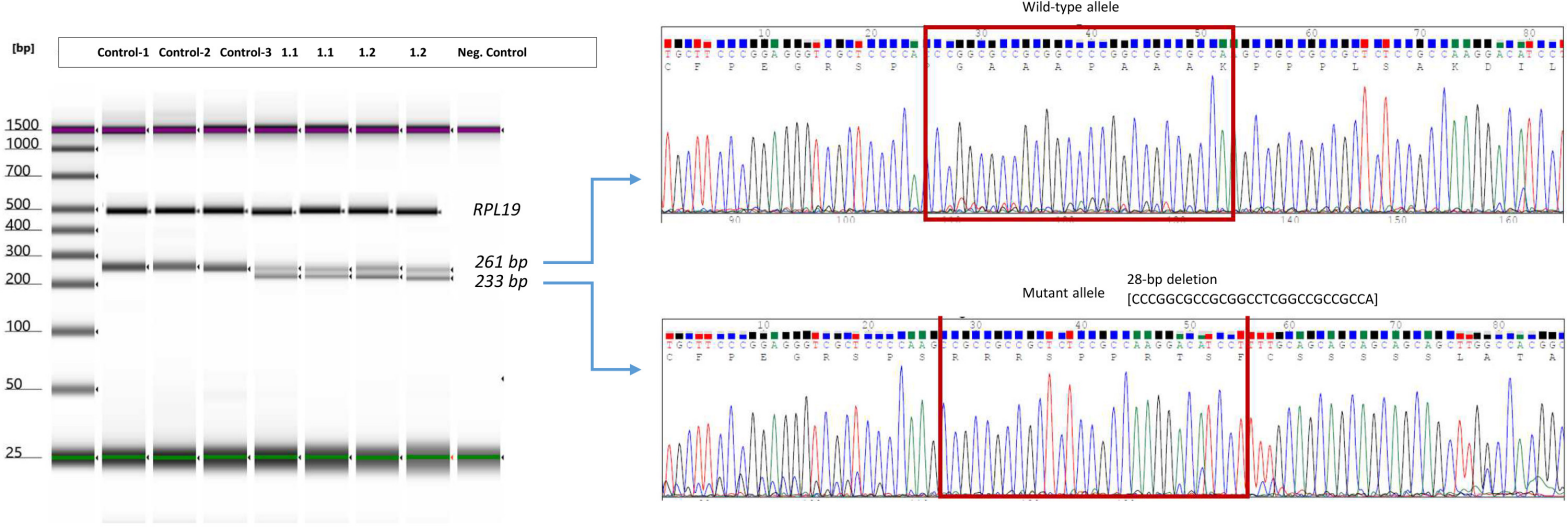
RT-PCR analysis of endogenous expression of IRF2BP2 in the patients from Family 1. Gel image of endogen expression of *RPL19* and *IRF2BP2* in three controls and affected patients from family 1. Amplification of wild-type allele and mutated allele by using specific primer design which allow to capture mutated alleles excluding wild-type sequence. Electropherogram of cDNA-sanger sequencing from patient 1.1 and 1.2 in exon 1 of *IRF2BP2 (*carriers of deletion of 28bp in the cDNA (c.217_244delACCCGGCGCCGCGGCCTCGGCCGCCGCC) is shown beside the PCR product in each case. The results were identical in both patients (see details in the **Supplementary Material**).

First, we corroborated the amplification of cDNA from an ubiquitously expressed endogenous gene, RPL19. Regarding IRF2BP2, the short PCR results revealed an additional band (233 bp) alongside the expected normal IRF2BP2 RT-PCR bands (261 bp) in both the proband’s father and daughter. Subsequent Sanger DNA sequencing confirmed that both the proband and the father carried the heterozygous deletion c.217_244del (**Figure 3**). This observation could not be precisely quantified, but we could estimate a semiquantitative value through cDNA concentration [ng/µl]. These data were consistent with half amount of cDNA of wild-type allele in patients 1.1 and 1.2 compared with the one observed in controls.

We performed an additional RT-PCR analysis to assess the endogenous expression of IRF2BP2 using allele-specific PCR assays targeting the c.991C>T polymorphism, located in the index case of family 1. Two separate PCR reactions were performed simultaneously: one with primers F1 and RWW to amplify cDNA allele C, and another with primers F1 and RWT to amplify cDNA allele T.

Once we amplified the fragment of exon 1, including the junction between exon 1 and exon 2, we proceeded to identify both wild-type allele and aberrant cDNA from the mutated allele. In patients 1.1 and 1.2, we were able to detect both wild-type and mutated alleles with the expected length, considering the deletion of 28 bp on the mutated allele (**Figure 3**). The expected band of the wild-type allele was 257 bp, whereas the mutated allele was expected to contain a fragment of 229 bp, considering a range of error of about 5 bp. Finally, we confirmed the amplification of cDNA in patients 1.1 and 1.2, performing Sanger sequencing from a PCR product obtained by amplification of specific oligonucleotides designed in the open-read-frame following the PCR assays.

### 3.4 Literature review of variants in IRF2BP2

**Figure 4:**
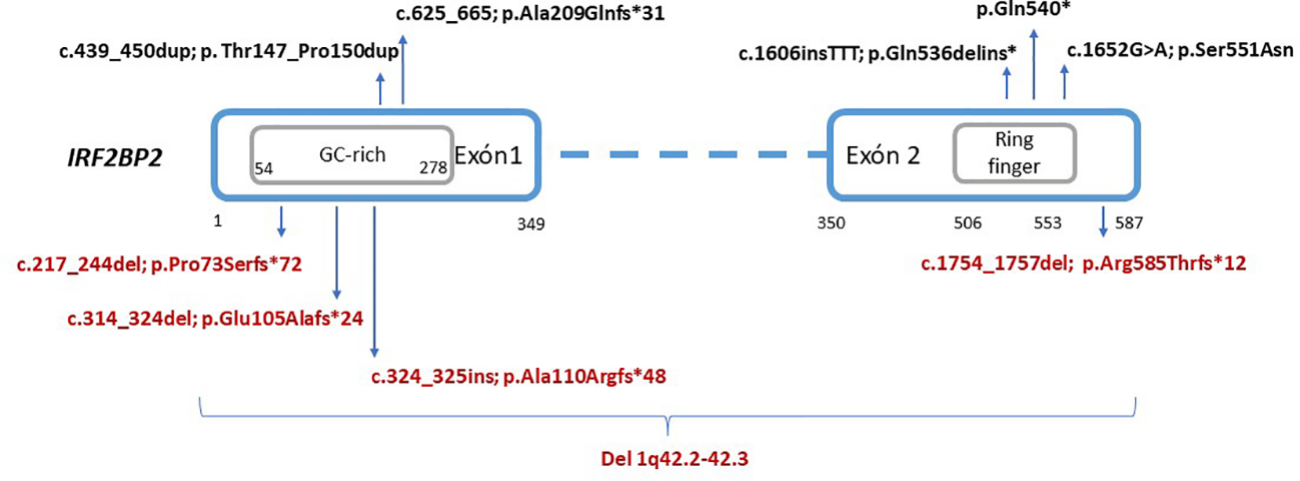
Schematic representation of reported variants in the *IRF2BP2* gene. The location of residues according to the domains and exons were extracted from the Nextprot database (https://www.nextprot.org/), whereas the GC-rich region is novelty estimated according to GC content. Variants in black (top) indicate previously reported variants. Variants in red (bottom) indicate novel variants reported in this study.

We search in several sources, mainly Pubmed articles, reported cases with disease-causing variants in IRF2BP2. For this purpose, we use key words such use “IRF2BP2”, “mutation/variant”. Only eight patients from five unrelated families harboring monoallelic pathogenic mutations in *IRF2BP2* have been previously reported (6, 11–14) (**Figure 4**). Both inherited and *de novo* events in the families evidence an autosomal dominant pattern with complete penetrance, nonetheless with limited data available in the scientific literature. The analytical features in almost all patients showed normal or decreased B-cell count with decreased memory B-cell isotype switching and severe hypogammaglobulinemia. However, expression and severity of the disease caused by LoF mutations in this gene are variable.

In addition to the eight reported cases, we present five additional cases, identified in five Spanish families which provide five novel LoF mutations in *IRF2BP2* (**Table 3**). Most of the patients started to develop symptoms during childhood, with the presence of recurrent respiratory infections, and were diagnosed with CVID in late adolescence or adulthood. Palmroth et al. (6) defined for the first time a different type of CVID with inflammatory manifestations due to disease-causing mutations in *IRF2BP2*. Inflammatory symptoms were observed in previous patients, mainly colitis and chronic diarrhea. Six out of eleven patients showed gastrointestinal involvement, with different degrees of severity. Notably, three families in this study manifested a variable phenotype including non-gastrointestinal autoinflammatory or autoimmune diseases such as RA, spondyloarthritis, severe eczema, hidradenitis suppurativa, myotonic dystrophy-2 or diabetes mellitus type 2. The patient 5.1 showed different phenotype and also the variant is still considered VUS because of the stop codon location.

**Table 3:** Phenotypic and genotypic features of known patients with disease-causing variants in IRF2BP2.

Family	Patient	Sex (age)	Variant	Zygosity	Inheritance	Infections	Diagnoses	Immuno- phenotype	Inflammatory Manifestations	Other	Reference
**A**	A.1	F (24)	p.Ser551Asn	Het	Autosomal dominant	Recurrent sinus infections since childhood	CVID	Normal B-cells. Decrease switched memory B-cells. Hypogamma-globulinemia, Poor vaccine responses	Colitis and chronic diarrhea	–	Keller et al., (11)
**A**	A.2 (index’s brother)	M (16*)	p.Ser551Asn	Het	Autosomal dominant	Chronic sinus infections	CVID	Normal B-cells. Hypogamma-globulinemia	–	–	Keller et al., (11)
**A**	A.3 (index’s father)	M (16)	p.Ser551Asn	Het	Autosomal dominant	Recurrent sinus infections	CVID	Normal B-cells. Decrease switched memory B-cells. Hypogamma-globulinemia	–	–	Keller et al., (11)
**B**	B.1	F (4)	p.Gln536delins*	Het	*De novo*	Recurrent infections	–	Hypogamma-globulinemia	Chronic diarrhea	Severe eczema, anemia, failure to thrive, fevers, short stature, cataracts, hypodontia, hypotrichosis alopecia	Baxter et al., 2021
**C**	C.1	M (57)	p.Ala209Glnfs*31	Het	Autosomal dominant	–	–	Lymphopenia, lymphadenopathy	Healing erosion in the duodenum, oral and genital ulcers, neutrophilic inflammation	Hidradenitis suppurativa, cholecystectomy, fatigue	Palmroth et al., (6)
**C**	C.2 (index’s sister)	F (71)	p.Ala209Glnfs*31	Het	Autosomal dominant	–	–	Lymphopenia	–	Hidradenitis suppurativa, DM2, hyperthyroidism, diabetes mellitus type 2	Palmroth et al., (6)
**D**	D.1	M (33)	p.Gln540*	Het	*De novo*	Recurrent respiratory infections	CVID	Severe decrease switched memory B-cells. Hypogamma-globulinemia, impaired CTLA4 expression in Treg.	Colitis, RA	–	Körholz et al., (13)
**E**	E.1	M (22)	p. Thr147_Pro150dup	Het	Autosomal dominant	Recurrent infections	Autoimmune encephalopathy		Crohn’s disease	Recurrent fever, psychiatric symptoms	Pan et al., (14)
**1**	1.1	F (42)	p.Pro73Serfs*72	Het	Autosomal dominant	Recurrent respiratory and gastrointestinal infections	CVID	Decrease B-cells, decrease switched of B-cells, hypogamma-globulinemia	Severe colitis	–	This study
**1**	1.2 (index’s father)	M	p.Pro73Serfs*72	Het	Autosomal dominant	Recurrent respiratory infections		Hypogamma-globulinemia,			This study
**2**	2.1	M (31)	p.Ala110Argfs*48	Het	*De novo*	Recurrent respiratory infections	CVID	Hypogamma-globulinemia,	Crohn’s disease. Intestinal resection	Spondylarthritis	This study
**3**	3.1	M	p.Glu105Alafs*24	Het	Autosomal dominant		CVID				This study
**4**	4.1	M	Del 1q42.2-42.3	Het	*De novo*	Recurrent respiratory infections	CVID	Hypogamma-globulinemia,	Recurrent bronchiectasis		This study
**5**	5.1	M	p.Arg585Thrfs*12	Het	Autosomal dominant	Recurrent varicella-zoster virus (VZV) infection, recurrent respiratory infections	–	Normal B-cells. Normal immunoglobulin values. Poor vaccine responses	–		This study

List of patients with LoF mutations in IRF2BP2. Families from A to E correspond to previously published patients. Families from 1 to 5 are reported in this study. CVID (common variable immunodeficiency, DM2 (dystrophia myotonica type 2), RA (rheumatoid arthritis).

## 4 Discussion

In this study, we report five novel variants in IRF2BP2 likely responsible of primary immunodeficiency, four of them were frameshift mutation and one a large CNV. The related phenotype of the patients described in this publication was compatible with primary antibody defects with variable expression since mild hypogammaglobulinemia and incomplete penetrance, to severe forms of CVID with enteropathy. We also highlight a non-described hotspot region of the gene with low complexity of sequence that predispose to accumulate indels changes.

The transcriptional corepressor protein IRF2BP2 contains 587 amino acids in the reference inform A, which isthe longest isoform (NM_182972.3; NP_892017.2). The aminoacidic sequence is organized into two exons, with one zinc finger RING-type domain located in the C-terminal region and, a nuclear localization signal in the N-terminal region. Considering the results of this study, we can highlight that a low-complex sequence with CG-rich, located in the first exon of *IRF2BP2*, may constitute a hotspot region susceptible to suffer insertions or deletions. When these variants lead to frameshift changes, their consequences are supposed to result in lack of protein expression and a pathogenic effect, considering that other pathogenic truncating mutations located in the exon 1 had been previously reported because of their LoF effect. In this sense, Palmroth et al. (6) constructed a plasmid with mutagenesis harboring c.625_665 (p.Ala209Glnfs*31) mutation in HEK293T cells, which expressed an aberrant IRF2BP2 protein of ~40Kda instead of the approximately 80-Kda wild-type protein, measured by immunoblotting (6). This assay demonstrated that truncating mutations in exon 1 disrupted the translation of the protein, but both this mutation and others previously reported truncating ones (p.Ala209Glnfs*31 and p.Gln540*) did no clarify whether these variants would suffer RNA degradation.

The findings reported in this study highlight non-degradation of RNA from mutated allele with c.217_244del (p.Pro73Serfs*72), suggesting that the variant escapes non-mediated mRNA decay (NMD). This mechanism has been previously proposed for nonsense variants located in exon 1, which are able to bypass NMD by using alternative methionine start codons (15). This event could explain why truncating mutations in exon 1 of *IRF2BP2* express mRNA, and it would also be a relevant aspect to further investigations about differences in the phenotype expression associated with genetic defects in *IRF2BP2*. These results also support the pathogenicity of truncating mutations in *IRF2BP2*, which are highly penetrant for CVID and show variable expression and severity of gastrointestinal manifestations, especially colitis, with autosomal dominant inheritance.

Regarding phenotype, patient 2.1, carrier of a *de novo* insertion (p.Ala110Argfs*48), suffered Crohn’s like enteropathy treated for a long time with anti-TNF therapy, and represents a good example of enteropathy associated with IRF2BP2-CVID. He later developed spondyloarthritis and finally underwent intestinal resection after the failure of the treatment. The depletion of *IRF2BP2* alters the responses to tumor necrosis factor α (TNF), affecting the signaling of inflammatory pathways (16). The variable severity of the disease may range from hypogammaglobulinemia with susceptibility to mainly respiratory infections to severe immunodeficiency with gastrointestinal inflammatory disease and autoimmune features. It is also remarkable that some individuals could be asymptomatic, indicating that there are forms with incomplete penetrance. In the patient 5.1, different phenotype was observed. Although one reason could possibly be explained by the distinct nature of the variant, we consider at this moment a VUS because of the predicted location of the stop codon, placed after the coding sequence of IRF2BP. This patient should be differently treated than cases with truncating variants, reported in this study, suggesting that the intrinsic mechanism of the variant p.Arg585Thrfs*12 did not cause the predicted phenotype for LoF variants in IRF2BP2. Consequently, further studies should be done to clarify the pathogenicity of this change.

From the technical point of view, the region encompassing nucleotides c.163_834 (p.54_278) is challenging to analyze by NGS because of its low complexity and CG-rich sequence, already highlighted since its description by Childs et al. (2003) (1). In fact, the sequence of *IRF2BP2* has an oncogenic role when it fuses with the *RARA* gene, previously reported in patients with promyelocytic leukemia (17). The recurrent variants found in the first exon of *IRF2BP2* in this study need to be confirmed by complementary techniques, consequently, improving the quality of the analysis of this gene. Otherwise, potential artefacts and low coverage regions may produce false negatives or positive results. In this context, very recently Pan et al., 2023 reported an in-frame insertion (c.439_450dup) in the low complexity region of IRF2BP2 in a patient with autoimmune encephalopathy and Crohn’s disease (14). We consider this variant a VUS, due to limitations of evidence about its pathogenicity. The in-frame changes in the low-complex region of exon 1 should be interpret with caution because of the susceptibility region to suffer mutational events. On the other hand, the expected phenotype of patients with LoF mutations in *IRF2BP2* include as major features CVID, hypogammaglobulinemia and other observed features include enteropathy and autoimmune manifestations. Atypical phenotypes in patients with genetic defects in this gene with different molecular mechanisms such as in-frame variants or extended codifying region of the gene need further studies to confirm their pathogenicity.

Before this study, only five families with CVID caused by defects in *IRF2BP2* had been published, now, we add five families from Spain with five novel truncating variants, which suggests that this gene may be undertested in the context of primary immunodeficiency and inflammatory conditions. Furthermore, this is the first time when a large *de novo* deletion involving the whole *IRF2BP2* gene is reported to cause of a condition associated with LoF. It is difficult to estimate the prevalence of this CVID subtype, but we can provide the data from an internal dataset based on our experience, observing 0,25% of patients with PID caused by defects in IRF2BP2 (>2000 patients), and approximately 5% of cases with suspected of CVID or primary defects of antibody (165 cases) with LoF mutations in *IRF2BP2.*

To the best of our knowledge, as observed in patients reported previously in the scientific literature and patients provided in this article, the examination of inflammatory symptoms as well as autoimmune features are important at the moment of diagnosis and also during follow-up of the disease in patients with genetic defects in *IRF2BP2*. Complete mechanisms in the regulation of IRF2BP2 expression are not fully understood; therefore, a better understanding of these mechanisms is needed to offers answers about different degrees of the disease severity. In fact, incomplete penetrance is not a rare phenomenon, as it was previously observed in other genetic defects in transcription factors associated with primary immunodeficiencies, such as IKZF1 or GATA3 (18, 19).

The results of this study were possible thanks to collect the data from 5 years of experience analyzing IRF2BP2 in patients with primary immunodeficiency. We provided the mRNA expression of one described variant in order to characterize the behavior of this mutation-type as a representative defect in exon 1. We only could perform this analysis in one variant, which was a limitation of this study. Further assays considering the protein effect and functional analysis on the biochemical pathways would provide mode precise data to evaluate the effect of this variants.

In conclusion, we describe four novel frameshift variants in IRF2BP2. Three of them were located in a delimited low-complex region enriched in GC nucleotides, which may be considered a hotspot for mutations in IRF2BP2. sequence. We describe the first large CNV in IRF2BP2 causing a likely new condition with CVID. The wide range of severity in this disorder make difficult the interpretation in the context of genetic counseling. It is very well established that the *IRF2BP2* gene should be included in genetic testing for CVID, accordingly it should be suspected and recommend *IRF2BP2*-gene screening in cases with severe colitis with recurrent respiratory and gastrointestinal infections. Regarding the involvement of IRF2BP2 in the repression of IRF2, it is expected that LoF leads to primary defects of adaptive immunity. However, IRF2BP2 also intervene in inflammatory processes, so we cannot discard those other phenotypes, different than primary defects of antibodies, may be associated with alterations in this gene. For this instance, it is important to evaluate de effect of the variants in this gene, specially when they are not predicted to cause LoF. It is still unknown the regulatory mechanisms that influence the expression of the gene, which may be a reason to explain the range of clinical manifestations and severity of the disease caused by LoF mutations in IRF2BP2.

## Data availability statement

The datasets presented in this study can be found in online repositories. The names of the repository/repositories and accession number(s) can be found below: https://www.ncbi.nlm.nih.gov/clinvar/variation/2577035/, https://www.ncbi.nlm.nih.gov/clinvar/variation/2577020/, https://www.ncbi.nlm.nih.gov/clinvar/variation/2577526/, https://www.ncbi.nlm.nih.gov/clinvar/variation/2577840/, https://www.ncbi.nlm.nih.gov/clinvar/variation/2578030/.

## Ethics statement

The studies involving humans were approved by COMITÉ ÉTICO DE INVESTIGACIÓN CLÍNICA DE CANTABRIA (CEIM - IDIVAL). The studies were conducted in accordance with the local legislation and institutional requirements. Written informed consent for participation in this study was provided by the participants’ legal guardians/next of kin. Written informed consent was obtained from the individual(s), and minor(s)’ legal guardian/next of kin, for the publication of any potentially identifiable images or data included in this article.

## Author contributions

JG-A: Conceptualization, Data curation, Formal Analysis, Investigation, Methodology, Supervision, Visualization, Writing – original draft, Writing – review & editing. EM: Formal Analysis, Investigation, Methodology, Writing – original draft. MR: Data curation, Writing – original draft. MA: Methodology, Writing – original draft. NP: Supervision, Writing – review & editing. LM: Supervision, Writing – review & editing. OC: Supervision, Writing – review & editing. PS-P: Supervision, Writing – review & editing. RC: Supervision, Writing – review & editing. EN: Supervision, Writing – review & editing. JO: Investigation, Supervision, Validation, Writing – review & editing.
