# Supplementary material for: Novel frameshift variants expand the map of the genetic defects in IRF2BP2
Source: Front Immunol. 2023 Oct 9;14:1279171. doi: 10.3389/fimmu.2023.1279171 (PMC10593445; doi:10.3389/fimmu.2023.1279171)
Supplement: Supplementary file 1 [file DataSheet_1.docx]

Supplementary Material

***S.1: Interpretation panel for immune system diseases.***

We used two cohorts of patients, from two hospitals (cohort 1:1783 patients; cohort 2: 318 patients). Each cohort of patients were studied by two different panels of genes associated with immune system diseases, both included IRF2BP2.

***Panel cohort 1: 549 genes***

*ABCB1, ACD, ACP5, ACTB, ADA, ADA2, ADAM17, ADAMTS13, ADAR, ADGRE2, AICDA, AIRE, AK2, ALPI, ALPK1, ANGPT1, ANXA11, AP1S3, AP3B1, AP3D1, APOL1, ARHGEF1, ARPC1B, ASAH1, ATG16L1, ATG4A, ATM, ATP6AP1, B2M, BACH2, BANK1, BCL10, BCL11B, BLK, BLM, BLNK, BLOC1S6, BTK, BTNL2, C17orf62, C1QA, C1QB, C1QC, C1QTNF4, C1R, C1S, C2, C2orf69, C3, C4A, C4B, C5, C6, C7, C8A, C8B, C8G, C9, CALCOCO2, CARD10, CARD11, CARD14, CARD8, CARD9, CARMIL2, CASP10, CASP8, CCBE1, CCDC88B, CCL2, CCR1, CCR3, CCR5, CCR9, CD14, CD19, CD226, CD247, CD27, CD36, CD3D, CD3E, CD3G, CD4, CD40, CD40LG, CD46, CD55, CD59, CD70, CD79A, CD79B, CD81, CD8A, CDC42, CDCA7, CEBPE, CFB, CFD, CFH, CFHR1, CFHR2, CFHR3, CFHR4, CFHR5, CFI, CFP, CFTR, CHD7, CIB1, CIITA, CLCN7, CLEC16A, CLEC7A, CLPB, COPA, CORO1A, CPT2, CR1, CR2, CSF2RA, CSF2RB, CSF3R, CTC1, CTLA4, CTNNBL1, CTPS1, CTSC, CXCL13, CXCR4, CXCR5, CYBA, CYBB, DBR1, DCLRE1B, DCLRE1C, DDX41, DDX58, DEF6, DGAT1, DGKE, DKC1, DNAJC21, DNASE1, DNASE1L3, DNASE2, DNMT3A, DNMT3B, DOCK2, DOCK8, EFL1, EGFR, EIF2AK2, ELANE, EPCAM, EPG5, ERAP1, ERBIN, ERCC6L2, ETS1, EXTL3, F12, FAAP24, FADD, FAS, FASLG, FAT4, FBLIM1, FCGR2A, FCGR2B, FCGR3A, FCGR3B, FCHO1, FCN3, FERMT1, FERMT3, FNIP1, FOXD3, FOXN1, FOXP3, FPR1, FUT2, G6PC3, G6PD, GATA1, GATA2, GFI1, GINS1, GUCY2C, HAS2, HAVCR2, HAX1, HELLS, HMOX1, HNF1A, HTRA2, HYOU1, ICAM1, ICOS, ICOSLG, IFIH1, IFNAR1, IFNAR2, IFNG, IFNGR1, IFNGR2, IGHM, IGKC, IGLL1, IKBKB, IKBKG, IKZF1, IKZF3, IL10, IL10RA, IL10RB, IL12A, IL12B, IL12RB1, IL12RB2, IL17F, IL17RA, IL17RC, IL18BP, IL18R1, IL1RN, IL21, IL21R, IL23A, IL23R, IL2RA, IL2RB, IL2RG, IL36RN, IL37, IL6, IL6R, IL6ST, IL7R, INAVA, INO80, IRAK1, IRAK4, IRF2BP2, IRF3, IRF4, IRF5, IRF7, IRF8, IRF9, IRGM, ISG15, ITCH, ITGAM, ITGB2, ITK, ITPKB, IVNS1ABP, JAGN1, JAK1, JAK2, JAK3, KDM6A, KIT, KLRC4, KMT2A, KMT2D, KNSTRN, KRAS, LACC1, LAMTOR2, LAT, LCK, LCP2, LIG1, LIG4, LIMK2, LPIN2, LRBA, LRP1, LRP5, LRRC8A, LSM11, LYST, MAGT1, MALT1, MAN2B1, MAP1LC3B2, MAP3K14, MAPK8, MASP1, MASP2, MBL2, MC2R, MC3R, MCM10, MCM2, MCM4, MECP2, MEFV, MICA, MICB, MKL1, MMACHC, MOGS, MPO, MS4A1, MSH6, MSN, MST1, MTHFD1, MVK, MYD88, MYH9, MYO5B, MYSM1, NBAS, NBN, NCF1, NCF2, NCF4, NCKAP1L, NCSTN, NEIL1, NEUROG3, NFAT5, NFE2L2, NFKB1, NFKB2, NFKBIA, NHEJ1, NHP2, NLRC4, NLRP1, NLRP12, NLRP3, NOD2, NOP10, NOS2, NPM1, NRAS, NSMCE3, OAS1, ORAI1, OSTM1, OTULIN, PARN, PAX1, PDCD1, PDGFRA, PEPD, PGM3, PHF11, PI4KA, PIK3CD, PIK3CG, PIK3R1, PLA2R1, PLCG2, PLEKHM1, PLG, PMS2, PNP, POLA1, POLD1, POLD2, POLE, POLE2, POLR3A, POLR3C, POLR3F, POMP, PRF1, PRKCD, PRKD1, PRKDC, PROC, PRPS1, PSENEN, PSMA3, PSMB10, PSMB3, PSMB4, PSMB8, PSMB9, PSMG2, PSTPIP1, PTEN, PTPN2, PTPN22, PTPRC, RAB27A, RAC2, RAG1, RAG2, RANBP2, RASGRP1, RBCK1, RC3H1, RECQL4, REL, RELA, RELB, RFX5, RFXANK, RFXAP, RHOH, RIPK1, RMRP, RNASEH2A, RNASEH2B, RNASEH2C, RNF168, RNF31, RNU4ATAC, RORC, RPSA, RTEL1, SAA1, SAMD9, SAMD9L, SAMHD1, SBDS, SEC61A1, SEMA3E, SERPING1, SGPL1, SH2D1A, SH3BP2, SH3KBP1, SHOC2, SIAE, SIK3, SKIV2L, SLC11A1, SLC26A3, SLC29A3, SLC35C1, SLC37A4, SLC39A7, SLC46A1, SLC7A7, SLC9A3, SMARCAL1, SMARCD2, SNX10, SOCS1, SP110, SPATA5, SPINK5, SPINT2, SPPL2A, SRP54, SRY, STAT1, STAT2, STAT3, STAT4, STAT5B, STAT6, STIM1, STK4, STN1, STX11, STXBP2, SYK, TAP1, TAP2, TAPBP, TAZ, TBK1, TBX1, TBX2, TBX21, TCF3, TCF7, TCIRG1, TCN2, TERC, TERT, TET2, TFRC, TGFB1, THBD, TICAM1, TINF2, TIRAP, TLR1, TLR2, TLR3, TLR5, TLR7, TLR8, TLR9, TMC6, TMC8, TMEM173, TNF, TNFAIP3, TNFRSF11A, TNFRSF13B, TNFRSF13C, TNFRSF1A, TNFRSF4, TNFRSF9, TNFSF11, TNFSF12, TNFSF13, TNFSF15, TNFSF4, TNIP1, TOM1, TOP2B, TPP2, TRAF1, TRAF2, TRAF3, TRAF3IP2, TREX1, TRIM21, TRIM22, TRNT1, TTC37, TTC7A, TYK2, UBA1, UBAC2, UBE2L3, UHRF1BP1, UNC119, UNC13D, UNC93B1, UNG, USB1, USP18, VPS13B, VPS45, VTN, VWA8, WAS, WDR1, WIPF1, WRAP53, XIAP, ZAP70, ZBTB24, ZNF341, ZNF699, ZNFX1.*

***Panel Cohort 2: 461 genes***

*ACD, ACP5, ACTB, ADA, ADA2, ADAM17, ADAMTS13, ADAR, AICDA, AIRE, AK2, ALPI, ANGPT1, AP1S3, AP3B1, AP3D1, APOL1, ARHGEF1, ARPC1B, ATAD3A, ATG4A, ATM, ATP6AP1, B2M, BACH2, BCL10, BCL11B, BLM, BLNK, BLOC1S6, BTK, C1QA, C1QB, C1QC, C1R, C1S, C2, C2orf69, C3, C4A, C4B, C5, C6, C7, C8A, C8B, C8G, C9, CARD11, CARD14, CARD9, CARMIL2, CASP10, CASP8, CCBE1, CD19, CD247, CD27, CD28, CD3D, CD3E, CD3G, CD40, CD40LG, CD46, CD48, CD55, CD59, CD70, CD79A, CD79B, CD81, CD8A, CDC42, CDCA7, CEBPE, CFB, CFD, CFH, CFHR1, CFHR2, CFHR3, CFHR4, CFHR5, CFI, CFP, CFTR, CHD7, CHUK, CIB1, CIITA, CLCN7, CLEC7A, CLPB, COPA, COPG1, CORO1A, CR2, CRACR2A, CSF2RA, CSF2RB, CSF3R, CTLA4, CTNNBL1, CTPS1, CTSC, CXCR2, CXCR4, CYBA, CYBB, CYBC1, DBF4, DBR1, DCLRE1B, DCLRE1C, DEF6, DGAT1, DGKE, DIAPH1, DKC1, DNAJC21, DNASE1L3, DNASE2, DNMT3B, DOCK2, DOCK8, EFL1, ELANE, ELF4, EPG5, ERBIN, ERCC6L2, EXTL3, F12, FAAP24, FADD, FAS, FASLG, FAT4, FCGR1A, FCGR2A, FCGR2B, FCGR3A, FCGR3B, FCGRT, FCHO1, FCN3, FERMT1, FERMT3, FNIP1, FOXN1, FOXP3, FPR1, G6PC3, G6PD, GATA2, GFI1, GINS1, HAVCR2, HAX1, HCK, HELLS, HMOX1, HS3ST6, HTR1A, HYOU1, ICOS, ICOSLG, IFIH1, IFNAR1, IFNAR2, IFNG, IFNGR1, IFNGR2, IGHM, IGKC, IGLL1, IKBKB, IKBKG, IKZF1, IKZF2, IKZF3, IL10, IL10RA, IL10RB, IL12B, IL12RB1, IL12RB2, IL17F, IL17RA, IL17RC, IL18BP, IL1RN, IL21, IL21R, IL23R, IL2RA, IL2RB, IL2RG, IL36RN, IL6R, IL6ST, IL7R, INO80, IRAK1, IRAK4****,*** *IRF2BP2, IRF3, IRF4, IRF7, IRF8, IRF9, ISG15, ITCH, ITGB2, ITK, ITPKB, ITPR3, JAGN1, JAK1, JAK2, JAK3, KDM6A, KMT2A, KMT2D, KNG1, KRAS, LACC1, LAMTOR2, LAT, LCK, LCP2, LIG1, LIG4, LPIN2, LRBA, LRRC8A, LSM11, LYST, MAGT1, MALT1, MAN2B2, MAP1LC3B2, MAP3K14, MAPK8, MASP2, MBL2, MCM10, MCM4, MEFV, MOGS, MPO, MRTFA, MS4A1, MSH6, MSN, MTHFD1, MVK, MYD88, MYOF, MYSM1, NBAS, NBN, NCF1, NCF2, NCF4, NCKAP1L, NCSTN, NEIL3, NFAT5, NFE2L2, NFKB1, NFKB2, NFKBIA, NFKBID, NHEJ1, NHP2, NLRC4, NLRP1, NLRP12, NLRP3, NOD2, NOP10, NOS2, NRAS, NSMCE3, OAS1, ORAI1, OSTM1, OTULIN, PARN, PAX1, PAX5, PDCD1, PEPD, PGM3, PIK3CD, PIK3CG, PIK3R1, PLCG2, PLEKHM1, PLG, PMS2, PNP, POLA1, POLD1, POLD2, POLE, POLE2, POLR3A, POLR3C, POLR3F, POMP, POU2AF1, PRF1, PRKCD, PRKDC, PSEN1, PSENEN, PSMA3, PSMB10, PSMB4, PSMB8, PSMB9, PSMG2, PSTPIP1, PTEN, PTPRC, RAB27A, RAC2, RAG1, RAG2, RANBP2, RASGRP1, RBCK1, RC3H1, RECQL4, REL, RELA, RELB, RFX5, RFXANK, RFXAP, RHOG, RHOH, RIPK1, RMRP, RNASEH2A, RNASEH2B, RNASEH2C, RNF168, RNF31, RNU4ATAC, RNU7-1, RORC, RPSA, RTEL1, SAMD9, SAMD9L, SAMHD1, SASH3, SBDS, SEC61A1, SEMA3E, SERPING1, SH2D1A, SH3BP2, SH3KBP1, SKIC2, SLC29A3, SLC35C1, SLC37A4, SLC39A7, SLC46A1, SLC7A7, SMARCAL1, SMARCD2, SNORA31, SNX10, SOCS1, SP110, SPI1, SPINK5, SPPL2A, SRP54, STAT1, STAT2, STAT3, STAT5B, STIM1, STK4, STN1, STX11, STXBP2, SYK, TAP1, TAP2, TAPBP, TAFAZZIN, TBK1, TBX1, TBX21, TCF3, TCIRG1, TCN2, TERC, TERT, TET2, TFRC, TGFB1, TGFBR1, TGFBR2, THBD, TICAM1, TINF2, TIRAP, TLR3, TLR7, TLR8, TMC6, TMC8, STING1, TNFAIP3, TNFRSF11A, TNFRSF13B, TNFRSF13C, TNFRSF1A, TNFRSF4, TNFRSF9, TNFSF11, TNFSF12, TNFSF13, TOM1, TOP2B, TPP2, TRAC, TRAF3, TRAF3IP2, TREX1, TRIM22, TRNT1, SKIC3, TTC7A, TYK2, UNC13D, UNC93B1, UNG, USB1, USP18, VPS13B, VPS45, WAS, WASF2, WDR1, WIPF1, WRAP53, XIAP, ZAP70, ZBTB24, ZNF341, ZNFX1*

***S.2: Sanger Sequencing of cDNA amplification from patient 1.1***

***A: Sanger Sequencing of cDNA of mutant allele carrier of the deletion.***


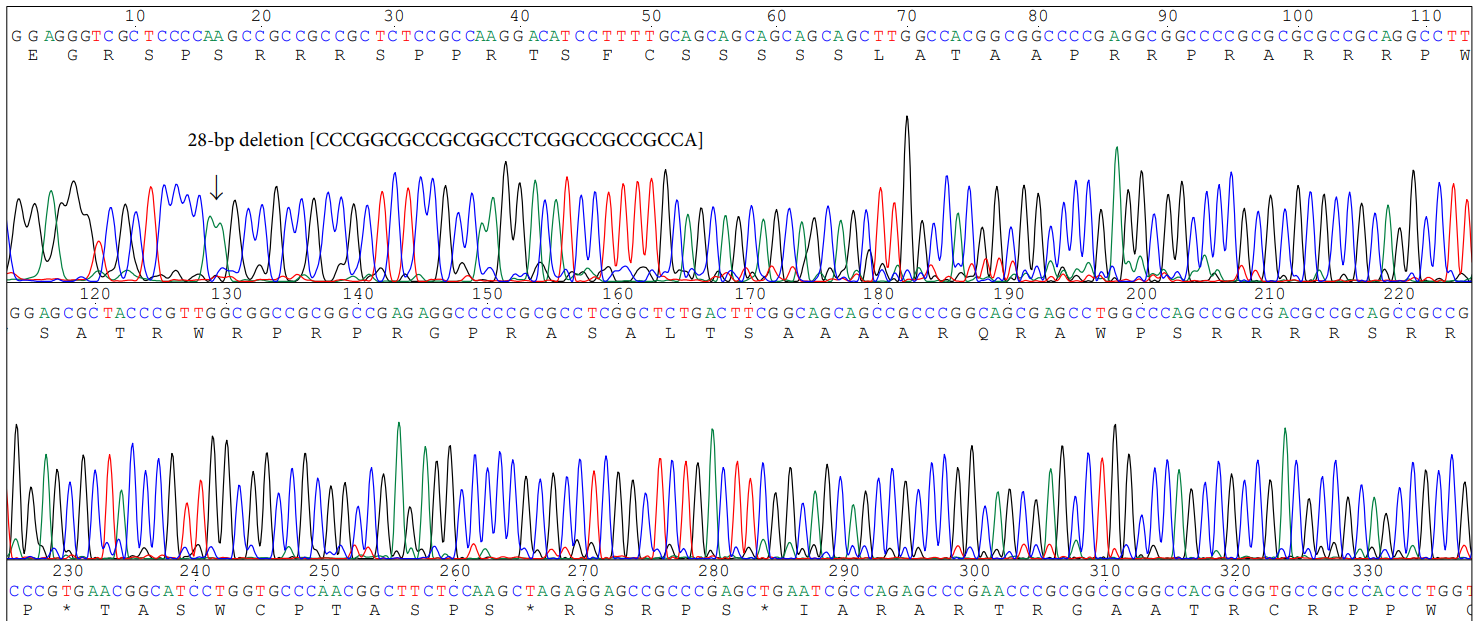


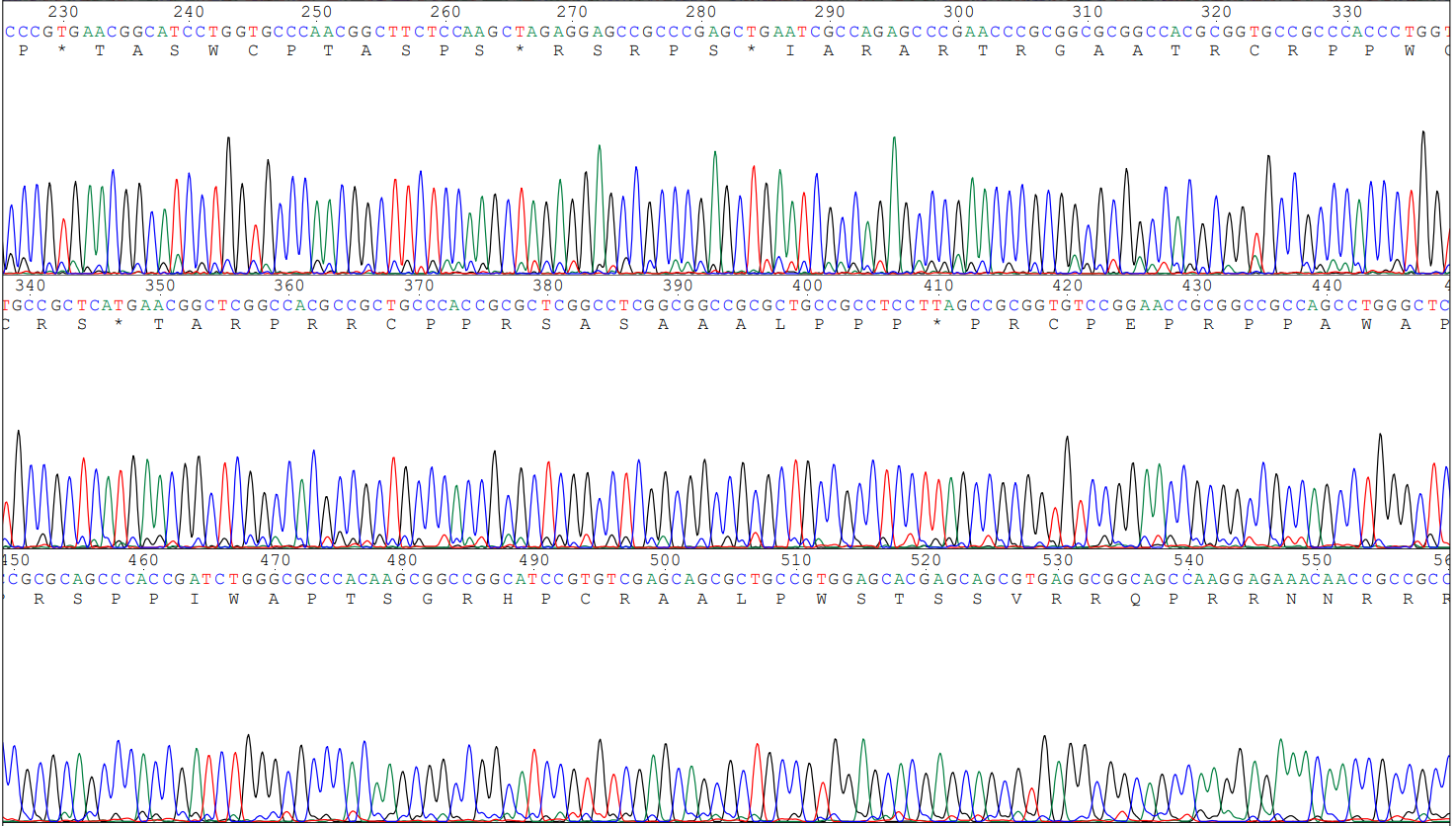


***B: Sanger Sequencing of cDNA of wild-type allele.***


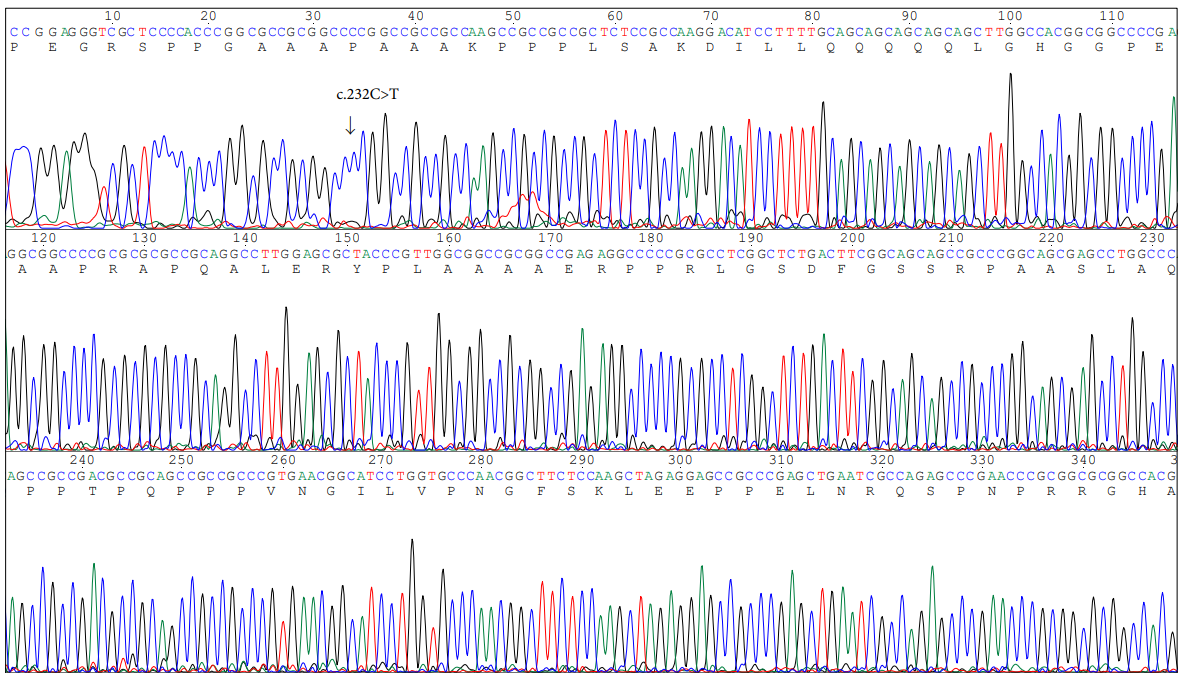


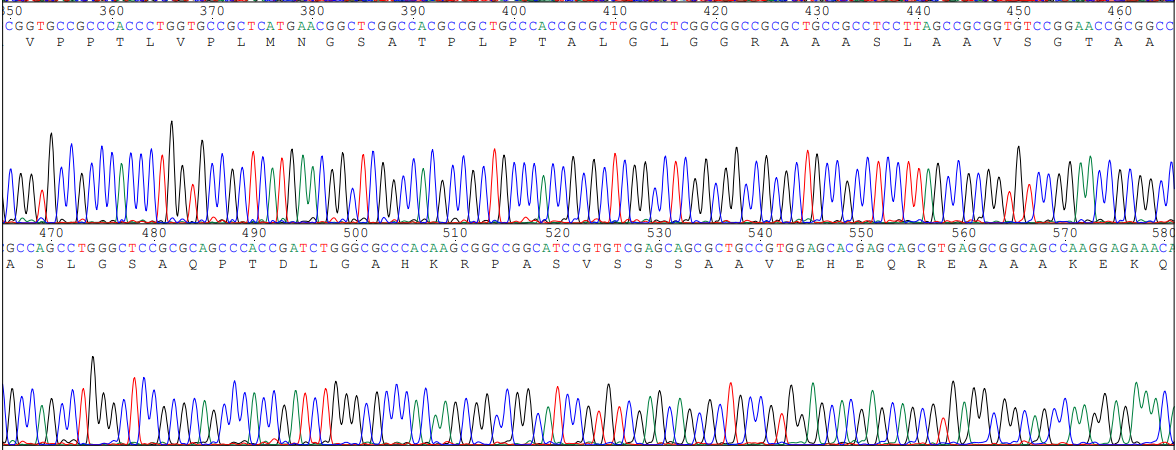


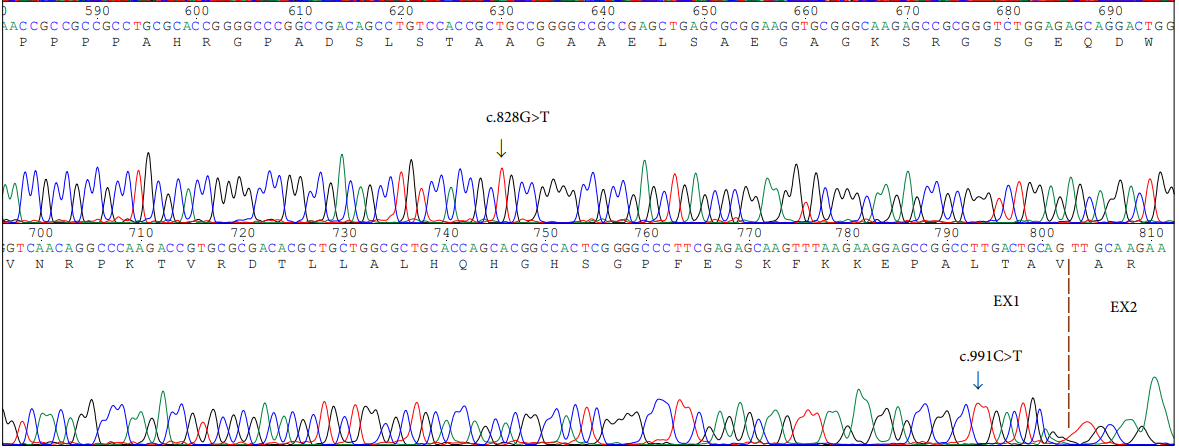


**
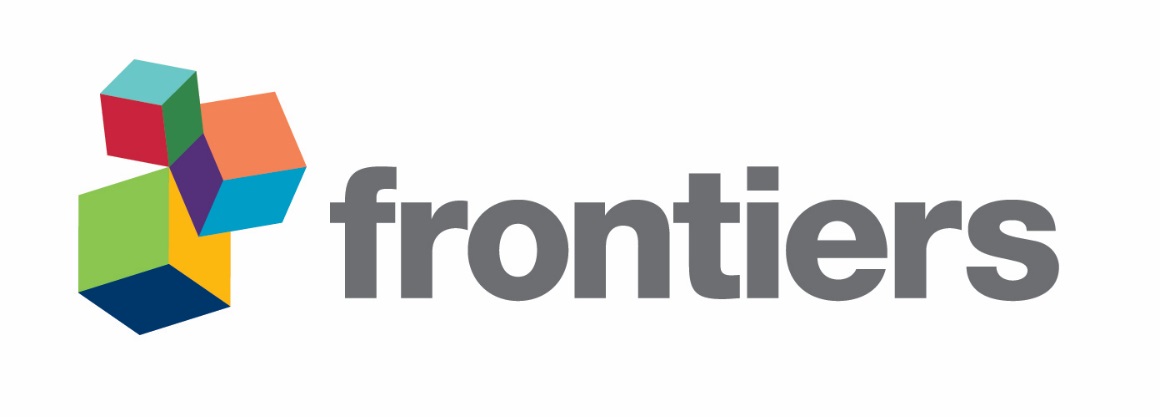
**
